# Anti-apoptotic activity of ET_B_ receptor agonist, IRL-1620, protects neural cells in rats with cerebral ischemia

**DOI:** 10.1038/s41598-019-46203-x

**Published:** 2019-07-18

**Authors:** Seema Briyal, Amaresh K. Ranjan, Mary G. Hornick, Anupama K. Puppala, Thanh Luu, Anil Gulati

**Affiliations:** 1 Chicago College of Pharmacy, Midwestern University, Downers Grove, IL 60515 USA; 2 Chicago College of Osteopathic Medicine, Midwestern University, Downers Grove, IL 60515 USA; 3 Present Address: Pharmazz, Inc., Research and Development, Willowbrook, IL USA

**Keywords:** Preclinical research, Stroke

## Abstract

Endothelin-B receptor agonist, IRL-1620, provides significant neuroprotection following cerebral ischemia in rats. Whether this neuroprotection is due to inhibition of apoptosis is unknown. IRL-1620-treated rats following permanent middle cerebral artery occlusion (MCAO) showed significant improvement in neurological and motor functions along with a decrease in infarct volume at 24 h (−81.3%) and day 7 (−73.0%) compared to vehicle group. Cerebral blood flow (CBF) significantly improved in IRL-1620-treated animals compared to vehicle by day 7 post MCAO. IRL-1620-treated rats showed an increase in phospho-Akt and decrease in Bad level 7 h post-occlusion compared to vehicle, while Akt and Bad expression was similar in cerebral hemispheres at 24 h post-MCAO. The phospho-Bad level was lower in vehicle- but not in IRL-1620-treated rats at 24 h. Anti-apoptotic Bcl-2 expression decreased, while pro-apoptotic Bax expression increased in vehicle-treated MCAO rats, these changes were attenuated (P < 0.01) by IRL-1620. Mitochondrial membrane-bound Bax intensity significantly decreased in IRL-1620 compared to vehicle-treated MCAO rats. IRL-1620 treatment reduced (P < 0.001) the number of TUNEL-positive cells compared to vehicle at 24 h and day 7 post MCAO. The results demonstrate that IRL-1620 is neuroprotective and attenuates neural damage following cerebral ischemia in rats by increasing CBF and reducing apoptosis.

## Introduction

Ischemic stroke is a significant worldwide health issue that can lead to serious long-term disability^1^. The pathophysiology behind ischemic stroke is multivariate and complex; therefore, developing an effective treatment capable of targeting multiple pathways is challenging. Prevention of apoptosis and restoration of blood flow are among the most important strategies, which could be target for treatment of ischemic stroke. Hence, a molecule or signaling pathway connected to neural cell survival/anti-apoptosis as well as blood perfusion would be an appropriate target. Since endothelin system is involved in the regulation of vascular tone as well as cell proliferation, survival and differentiation^2–5^, it may be an important target to treat stroke.

Endothelin (ET), an endogenous 21 amino acid peptide, produces its biological effects through activation of G-protein-coupled receptors: ET_A_ and ET_B_^6,7^. Interestingly, the expression of endothelin B (ET_B_) receptor has been observed on vascular cells as well as neural cells in the central nervous system (CNS) and plays important roles in cell survival and proliferation^8–12^. Moreover, the role of ET_B_ receptors in the development of the CNS has been demonstrated by using homozygous ET_B_ receptor deficient rats (spotting lethal rats, sl/sl) and a transgenic ET_B_ receptor deficient rescue rats in prenatal and postnatal stages, respectively^9,13,14^. CNS disturbances and fatal birth defects are noted when ET_B_ receptors are deficient or absent during the prenatal period. ET_B_-deficient rats have been shown to present with a decrease in neuronal progenitors and an increase in apoptosis within the dentate gyrus and cerebellum^9,14^, while the ET_B_ receptor knockout model is characterized by significant CNS disturbances and proves fatal within 4 weeks of birth^13,15^. Ontological studies have demonstrated the integral function of ET_B_ receptors within the developing CNS, promoting neuronal proliferation and migration as well as angiogenic growth factors^14,16^. ET_B_ receptors play a crucial role not only in the developmental stage of the CNS, but their stimulation following CNS damage in adults has also been shown to enhance neurogenesis and angiogenesis, thereby promoting CNS repair and regeneration^17–19^.

Previous studies from our laboratory have demonstrated that selective stimulation of ET_B_ receptors by agonist, IRL-1620, could significantly improve neurological and motor functions, with concurrent decrease in infarct volume and oxidative stress damage following permanent middle cerebral artery occlusion (MCAO) in rats^20,21^. IRL-1620 treatment post cerebral ischemia has been shown to protect neurons while enhancing angiogenesis, as noted by an increase in both neuronal nuclei and vascular endothelial growth factor (VEGF). Additionally, animals receiving IRL-1620 displayed increased numbers of proliferating cells and cells positively staining for nerve growth factor (NGF) in the infarcted brain^22^. Recently, a clinical trial phase I was conducted and IRL-1620 was found to be safe and well tolerated in healthy human volunteers (CTRI/2016/11/007509)^23^. A randomized, double blind, controlled, multicenter phase II trial is in progress in patients with cerebral ischemic stroke (CTRI/2017/11/010654).

Our studies indicate the neuroprotective and neurorestorative roles of ET_B_ receptor stimulation by IRL-1620 following cerebral ischemia. However, anti-apoptotic pathway involved in IRL-1620’s neuroprotective effects after ischemia remain to be identified. Neuronal apoptosis is a significant factor in neurological disorders associated with ischemia. Anti-apoptotic mechanisms in the rat cortical neurons have been shown to be positively affected by ET_B_ receptor agonists^24,25^. Increased neuronal apoptosis has been observed in areas containing progenitor cells of spotting-lethal (sl/sl) rats lacking functional ET_B_ receptors^9,13^.

In the present study, cerebral ischemia was induced in rats by permanent MCAO, and then they were treated with vehicle or IRL-1620 (5 µg/kg, i.v.) at 2, 4, and 6 h post-occlusion. We determined cerebral blood flow using laser Doppler flow meter before, 1 h and on day 7 post MCAO. Behavioral parameters and analysis of cell survival and apoptotic pathways markers e.g. Akt, pAkt, Bcl-2, Bad and Bax (cytosolic and mitochondrial membrane-bound) were carried out  in sham, IRL-1620 and vehicle-treated MCAO rats at 7 h, 24 h and day 7 post occlusion. We also assessed the DNA fragmentation (a hallmark of the end stage apoptosis) by TUNEL assay.

## Results

### IRL-1620 treatment alleviates neurological deficit in MCAO rats

Seven hours, 24 h and day 7 after MCAO of the right side, paresis of the left hind limb was observed. No difference in the mean neurological score of vehicle and IRL-1620-treated animal was observed at 7 h post MCAO. At 24 h and Day 7 post MCAO, compared to sham-operated rats, the mean neurological score of vehicle-treated rats was significantly higher (P < 0.001), indicative of neurological impairment following induction of cerebral ischemia. In contrast, MCAO rats treated with IRL-1620 demonstrated significant improvement in neurological function when compared with the vehicle group (P < 0.001). (Table 1).

**Table 1:** Effect of ET_B_ receptor agonist, IRL-1620, on neurological deficit and motor functions at baseline, 7 h and 24 h post MCAO in rats.

Treatment Groups	Time points	Neurological deficit (6 point scale)	Grip test (6 point scale)	Foot fault error (%)	Rota rod duration (Sec)	Distance traveled (cm)
Sham	Baseline	0 ± 0	5 ± 0	2.8 ± 1.3	133.2 ± 7.3	3957.8 ± 464.9
	7 h post MCAO	0 ± 0	4.2 ± 0.3	2.8 ± 0.3	143.2 ± 14.5	3446.6 ± 296.6
	24 h Post MCAO	0 ± 0	4.8 ± 0.2	9.1 ± 0.4	183.1 ± 9.9	3758.2 ± 265.9
	Day 7 post MCAO	0 ± 0	3.6 ± 0.2	5.5 ± 1.5	142.8 ± 18.7	4160.8 ± 501.8
MCAO + Vehicle	Baseline	0 ± 0	4.2 ± 0.2	2.4 ± 1.5	134.8 ± 7.8	3901.8 ± 472.3
	7 h post MCAO	2.5 ± 0.2*	1.5 ± 0.7*	58.5 ± 14.9*	64.0 ± 8.0*	543.6 ± 170.2*
	24 h Post MCAO	4.0 ± 0.3*	0.5 ± 0.2*	86.0 ± 4.0*	43.0 ± 5.8*	269.3 ± 129.6*
	Day 7 Post MCAO	2.3 ± 0.2*	1.5 ± 0.4*	44.3 ± 8.1*	113.7 ± 19.6	2882.0 ± 249.6*
MCAO + IRL-1620	Baseline	0 ± 0	4.5 ± 0.2	2.2 ± 1.4	122.5 ± 12.0	4010.3 ± 312.3
	7 h post MCAO	1.8 ± 0.4	2.1 ± 0.5*	39.9 ± 5.8*	112.0 ± 35.0	1275.6 ± 293.8*
	24 h Post MCAO	1.8 ± 0.2^#^	3.5 ± 0.2^#^	19.6 ± 3.8^#^	125.1 ± 7.3^#^	3526.2 ± 256.3^#@^
	Day 7 post MCAO	0.8 ± 0.1*^#^	4.4 ± 0.3^#^	11.8 ± 4.4^#^	163.4 ± 22.8^#^	3782.7 ± 552.8^#@^

Values are expressed as mean ± S.E.M. *P < 0.001 compared to sham. ^#^P < 0.01 compared to MCAO + vehicle. ^@^P < 0.05 compared to 7 h post MCAO + IRL-1620.

### IRL-1620 treatment improves motor function and coordination in MCAO rats

While there was no difference in motor function or coordination in any of the animals prior to occlusion, significant impairment was noted 7 h, 24 h and day 7 after induction of cerebral ischemia. No significant difference was observed in functional parameters between vehicle and IRL-1620-treated animal at 7 h post MCAO (Table 1).

#### Grip test

Vehicle-treated rats demonstrated a significant decrease in muscle strength following MCAO compared to sham-operated rats (P < 0.001). In contrast, rats treated with IRL-1620 showed significant improvement (P < 0.001) when compared with vehicle-treated rats, with mean scores of 3.5 ± 0.2 vs. 0.5 ± 0.2, respectively 24 h post MCAO and 4.4 ± 0.3 vs. 1.5 ± 0.4, respectively day 7 post MCAO (Table 1).

#### Foot fault

A less than 10% foot fault error was observed prior to occlusion. After MCAO, a lack of motor coordination was found in vehicle-treated animals, with this group of animals having a significantly higher percentage of foot fault errors compared to sham (P < 0.001). Treatment with IRL-1620, resulted in improved coordination, with a significantly lower percentage of error as compared with vehicle-treated rats (19.6 ± 3.8% vs. 86.0 ± 4.0% at 24 h; 11.8 ± 4.4% vs. 44.3 ± 8.1% at day 7; P < 0.01) (Table 1).

#### Rota rod

After occlusion, vehicle-treated MCAO rats demonstrated poor coordination by failing to remain on the rod when compared with sham-operated animals (P < 0.001). IRL-1620-treated occluded rats demonstrated significant improvement in motor coordination (125.1 ± 7.3 s and 163.4 ± 22.8 s at 24 h and day 7, respectively) compared with vehicle-treated rats (43.0 ± 5.8 s and 113.7 ± 19.6 s; P < 0.01) (Table 1).

#### Locomotor activity

Spontaneous motor activity was comparable in all groups prior to occlusion. Following MCAO, vehicle-treated rats were moving significantly (P < 0.001) less than the sham-operated. However, when IRL-1620 was administered, spontaneous activity was significantly (p < 0.01) increased (Table 1).

### IRL-1620 increases phosphorylation of Akt transiently and decreases Bad expression

**Figure 1:**
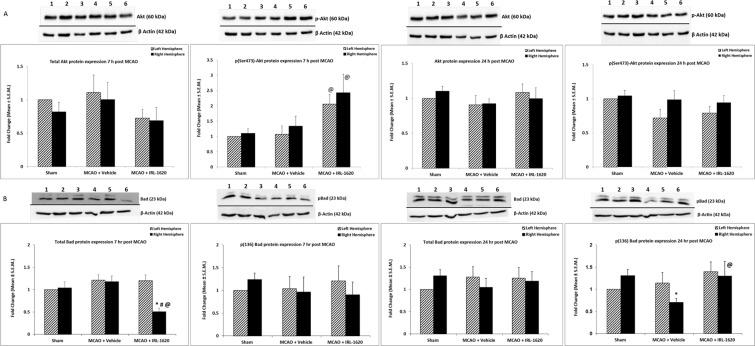
Expression of total Akt and pAkt (**A**) and total Bad and pBad (**B**) protein levels with β-actin as a loading control. Lane 1- Sham [LH]; Lane 2 – Sham [RH]; Lane 3 –MCAO + Vehicle [LH]; Lane 4 –MCAO + Vehicle [RH]; Lane 5 – MCAO + IRL-1620 [LH]; Lane 5 – MCAO + IRL-1620 [RH]. LH = Left hemisphere; RH = Right hemisphere. *P < 0.01 compared to sham, ^#^P < 0.05 compared LH; ^@^P < 0.001 compared to MCAO + vehicle [RH]. Full-length blots are presented in supplementary file.

We observed a significant increase in the phosphorylation of Ser473 of Akt (pAkt) in right (occluded) hemisphere of IRL-1620 treated rats compared to the sham (P < 0.01) and vehicle-treated (P < 0.05) rats 7 h post MCAO; however, no significant difference in pAkt level was seen among the groups at 24 h post MCAO. Moreover, no significant change was noticed in total Akt expression among these groups at 7 h and 24 h post MCAO (Fig. 1A).

We also examined the expression of the pro-apoptotic protein, Bad at 7 h and 24 h post MCAO in these samples. At 7 h post MCAO, a significant decrease in total Bad expression was observed in the right (occluded) hemisphere of the IRL-1620-treated rats compared to the sham and vehicle-treated groups (P < 0.05) as well as compared to the contralateral hemisphere of the IRL-1620-treated rats (P < 0.05). However, no change in total Bad expression was observed at 24 h post MCAO. We also assessed the level of phospho-Bad (inactive form) at 7 h and 24 h post MCAO. There was no difference in the phosphorylation of the Ser136 residue of Bad (pBad) 7 h post MCAO; however, a decreased level of phospho-Bad (pBad) was observed in the occluded hemisphere of the vehicle-treated group compared to the sham and IRL-1620-treated rats (P < 0.05) 24 h post MCAO (Fig. 1B).

### IRL-1620 upregulates anti-apoptotic Bcl-2 and downregulates pro-apoptotic Bax expression

**Figure 2:**
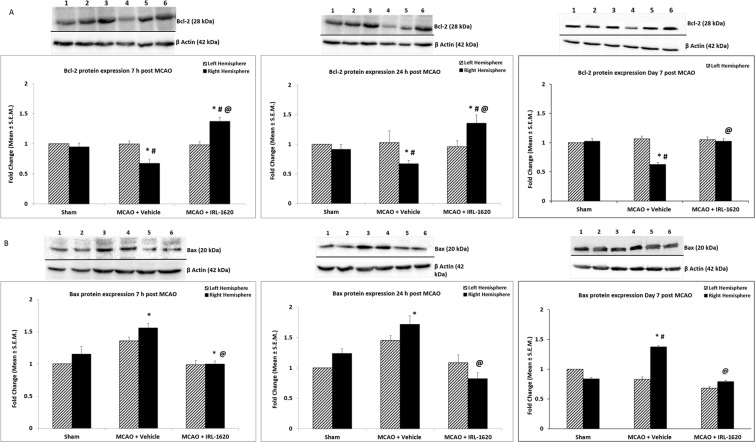
Expression of Bcl-2 (A) and Bax (B) protein levels with β-actin as a loading control. Lane 1 – sham [LH]; lane 2 – sham [RH]; lane 3 –MCAO + Vehicle [LH]; lane 4 –MCAO + vehicle [RH]; lane 5 –MCAO + IRL-1620 [LH]; lane 6 –MCAO + IRL-1620 [RH]. Values are expressed as mean ± S.E.M. *P < 0.01 compared to sham, ^#^P < 0.05 compared LH; ^@^P < 0.001 compare to MCAO + vehicle [RH]. Full-length blots are presented in supplementary file.

In the right (occluded) hemisphere of the vehicle-treated rats, expression of Bcl-2 was decreased at 7 h, 24 h and day 7 post MCAO compared to sham (p < 0.001). On the other hand, in IRL-1620-treated rats Bcl-2 expression was increased in the occluded hemisphere compared to vehicle-treated rats (P < 0.001) at 7 h, 24 h and day 7 post MCAO. In comparison to sham at 7 h and 24 h post-surgery, the Bcl-2 expression in IRL-1620-treated animals was also significantly (P < 0.05) increased (Fig. 2A). As expected, pro-apoptotic Bax expression was upregulated in vehicle-treated animals in response to ischemia compared to sham; however, IRL-1620 treatment significantly attenuated the ischemia-induced increase in Bax expression (P < 0.001) at 7 h, 24 h and day 7 post MCAO (Fig. 2B).

**Figure 3:**
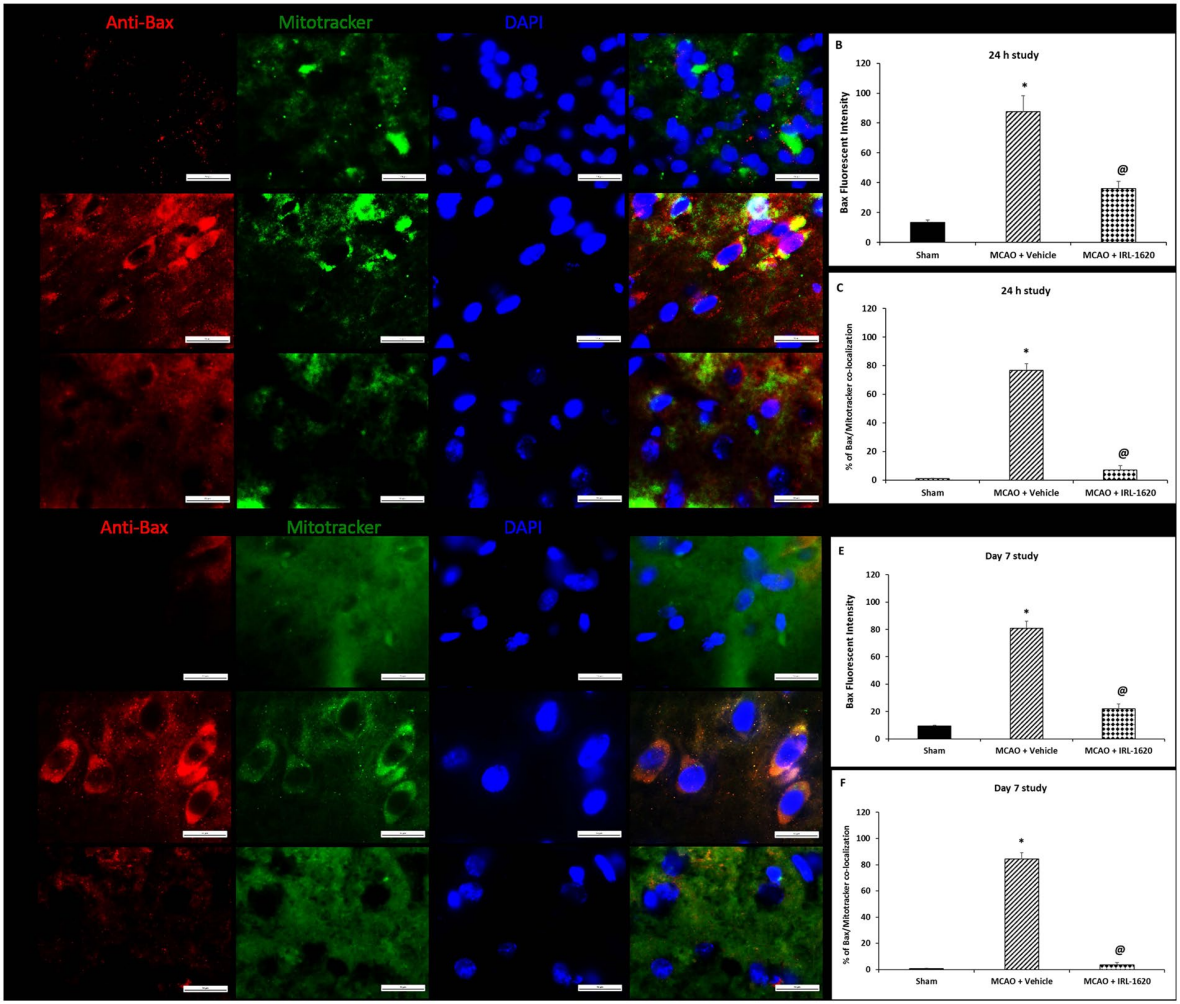
Bax translocation to mitochondria in cerebral ischemia-induced apoptosis. Bax was immuno-stained with anti-Bax^34^, and the mitochondria were stained with MitoTracker (green). The merged image indicates colocalization of Bax on mitochondria. Values are expressed as mean ± S.E.M. *P < 0.01 compared to sham, ^@^P < 0.001 compare to MCAO + vehicle.

The Bcl-2 family proteins are known as an important gatekeeper to the apoptotic response in the neuronal mitochondria-pathway^26^. Following a death stimulus, cytosolic and monomeric Bax proteins change to homo-oligomeric form and translocate to the mitochondrial outer membrane and assist the flow of apoptotic factors to the cytosol^27,28^. Thus, integration of Bax into the mitochondrial membrane is a critical step of apoptosis from where the reversal of apoptosis is highly unlikely. Therefore, detection of mitochondrial membrane-bound Bax would be a highly relevant indicator of apoptosis than cytosolic alone. We used immunofluorescence technique to detect the co-localization of Bax and mitochondria in the rat brain tissues after staining with anti-Bax and mitotracker dye. We observed very low levels of Bax expression in sham but significantly higher expression in vehicle-treated animals at both 24 h (Fig. 3A,B) and day 7 post MCAO (Fig. 3D,E). Treatment with IRL-1620 decreased the Bax expression significantly compared to the vehicle group (Fig. 3), which confirm the results obtained from western blot (Fig. 2B). Interestingly, when we analyzed the microscopic images for co-localization of Bax and mitochondria, we observed a higher amount of Bax co-localization with mitotracker dye in the vehicle group compared to IRL-1620-treated animals at both 24 h (Fig. 3A,C) and day 7 post MCAO (Fig. 3D,F).

### IRL-1620 treatment decreases DNA fragmentation

**Figure 4:**
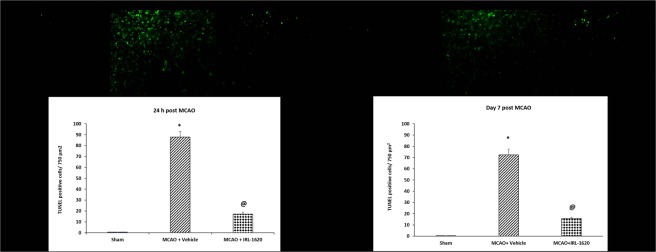
TUNEL positive cells per 750 µm^2^ in the ischemic region were detected by TUNEL staining 24 h and day 7 after MCAO. Values are expressed as mean ± S.E.M. *p < 0.0001 compared to sham; ^@^p < 0.001 compared to vehicle.

TUNEL assay was employed to study the effect of IRL-1620 on DNA fragmentation at 24 h and day 7 after MCAO. A significant amount of TUNEL-positive cells at 24 h and day 7 post MCAO were detected in the vehicle-treated group (87.9 ± 5.2 and 72.4 ± 4.3 per 750 µm^2^, respectively) as compared with the sham-operated group. After treatment with IRL-1620, the level of DNA fragmentation in the ischemic hemisphere was significantly decreased at 24 h and day 7 (17.2 ± 1.6 and 15.6 ± 1.3 per 750 µm^2^, respectively, P < 0.001) when compared to the vehicle group (Fig. 4).

### IRL-1620 treatment improves cerebral blood flow (CBF) and decreases infarct volume in MCAO rats

**Figure 5:**
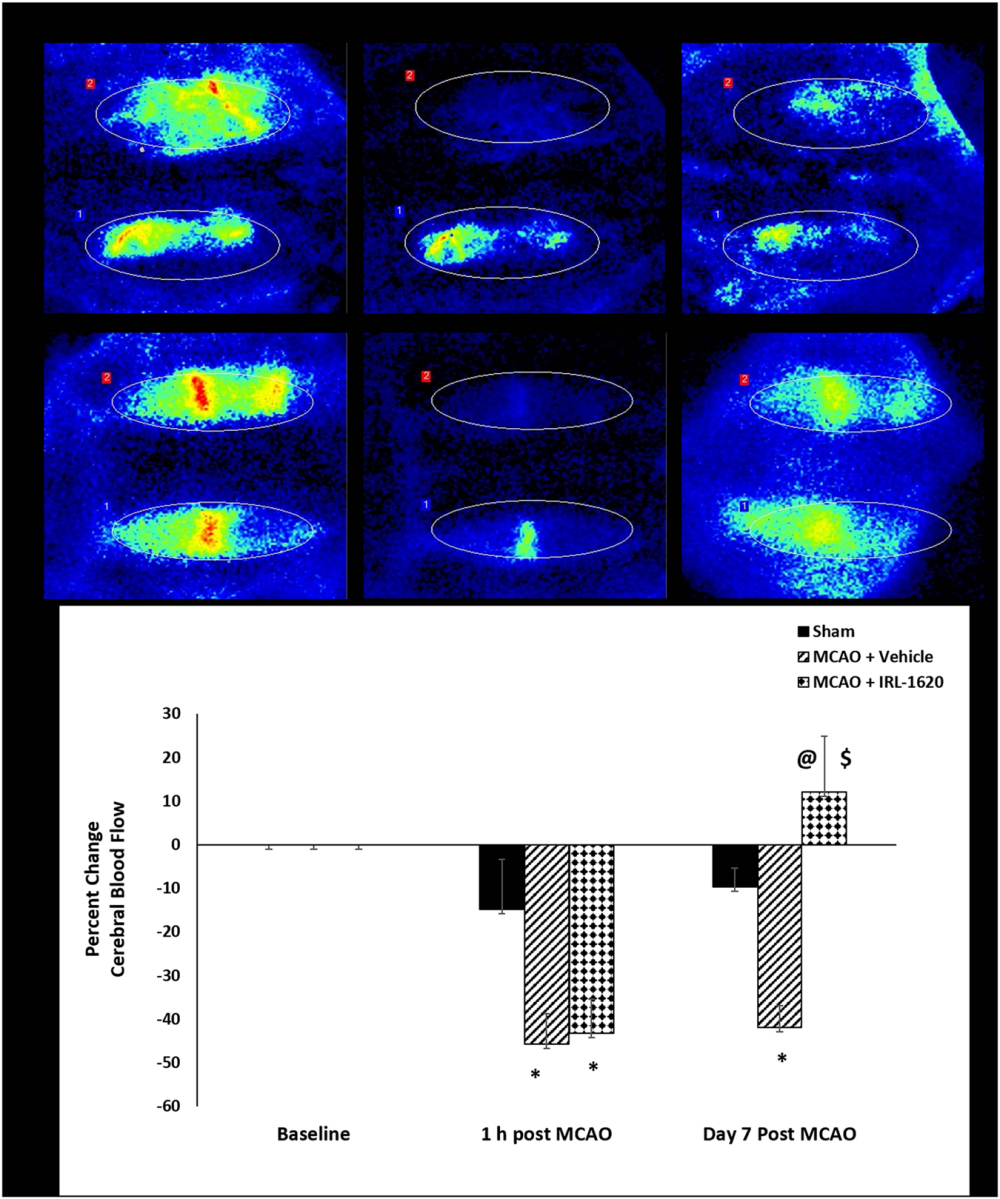
Effect of IRL-1620 on cerebral blood flow before, after and day 7 post MCAO in rat brains. Values are expressed as mean ± SEM. *P < 0.001 compared to sham; ^@^P < 0.05 compared to MCAO + vehicle; ^$^P < 0.0001 compared to IRL-1620 1 h post MCAO.

We assessed the CBF 15 min prior to MCAO (baseline), 1 h and day 7 post MCAO. There was 40–45% reduction in CBF in the occluded hemisphere compared to baseline at 1 h post MCAO. CBF in the vehicle-treated group remained unchanged (−41.9 ± 5.0%, below baseline), whereas CBF significantly (p < 0.001) improved in IRL-1620-treated animals (+12.0 ± 12.9%, above baseline) after day 7 post MCAO (Fig. 5).

**Figure 6:**
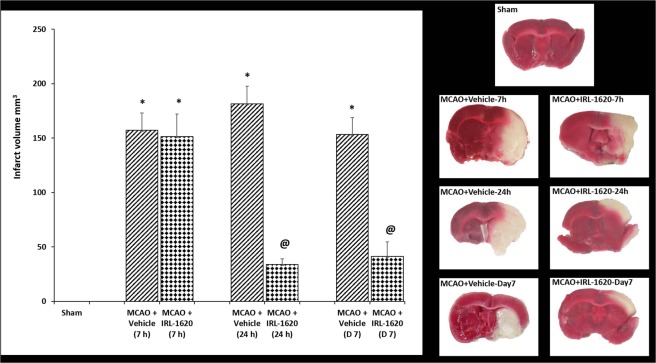
Effect of IRL-1620 on infarct volume in MCAO rats. 2 mm coronal sections of brains stained with TTC to visualize the infarct area 7 h, 24 h and day 7 post MCAO (red indicates normal tissue and white indicates infarct tissue). Values are expressed as mean ± SEM. *P < 0.001 compared to sham; ^@^P < 0.05 compared to MCAO + vehicle.

There was no significant difference in the infarct volume of vehicle and IRL-1620-treated animal at 7 h post MCAO (157.1 ± 15.9 and 151.3 ± 20.7 mm^3^, respectively). Cerebral ischemia post MCAO resulted in an infarct volume of 181.6 ± 16.0 and 153.4 ± 15.5 mm^3^ in rats treated with vehicle at 24 h and day 7, respectively. IRL-1620 treatment significantly decreased the infarct volume at 24 h and day 7 post MCAO (33.9 ± 5.1 and 41.4 ± 13.4 mm^3^, respectively; P < 0.001) compared to vehicle treated animals (Fig. 6).

## Discussion

We have demonstrated that selective stimulation of the ET_B_ receptors enhances neurovascular repair with significant reductions in neurological deficit, infarct volume and oxidative stress in rat models of cerebral ischemia^20–22^. We also observed higher cell proliferation (BrdU^+^ cells) in IRL-1620-treated rat brains compared to control^22^, however its role in protection of cells against apoptotic damage during stroke remained elusive. Since the inhibition of apoptotic cell death has been reported as an important event in the neuroprotection^29,30^, we conducted this study to determine whether ET_B_ receptor stimulation also affects the apoptotic pathways following ischemia. We used a well-established method of permanent middle cerebral artery occlusion (MCAO) in rats to induce ischemia in the brain. We have been regularly using this procedure in our lab for more than 10 years and produces highly consistent results confirmed by measuring CBF using laser Doppler flow meter. It involves occlusion of MCA, which is most often affected in human ischemic stroke and accounts for ~70% of infarcts^31^. Thus, the MCAO technique is the closest to human ischemic stroke with affecting similar regions of the brain in animal models. Other advantages of the MCAO model include large infarct volume and high reproducibility. It has been demonstrated that the MCAO technique is the most preferred technique for reproducing ischemic stroke, as it results in neural cell death, inflammation, damage to the blood brain barrier as well as producing dependable results in behavior tests. Therefore, among the occlusive models for stroke the MCAO technique is the model of choice and has been used in more than 40% of all the experiments (~2600) on neuroprotection after ischemic stroke^32^. After successful MCAO, the rats were randomly selected for injection of either IRL-1620 (5 µg/kg) or saline (vehicle) at 2, 4 and 6 h post occlusion. The dose of IRL-1620 was based on our previous studies^20–22^ and Phase I safety and tolerability studies, where the Minimum Intolerable Dose (MID) for IRL-1620 was established as 0.9 μg/kg and the Maximum Tolerated Dose (MTD) was 0.6 μg/kg (CTRI/2016/11/007509)^23^. According to dose by factor method the dose of 0.6 μg/kg (MTD) in human is approximately equivalent to 5 μg/kg in rats^33^. The half-life of IRL-1620 ranges from 4.38 min to 8.29 min^34^. The observed half-life of IRL-1620 is short; however, a longer duration of action is possible similar to the ET-1 signaling, where ET-1 and its receptors get internalized within 10 min and continues to signal for days^35–37^.

We sacrificed rats at 7 h, 24 h and day 7 post MCAO for analysis of different parameters. The analyses of cerebral blood flow (noninvasive) at 1 h and infarct size measurement at 7 h post occlusion showed similar blood flow reduction and infarct size in both groups, which confirmed the consistency of the effect of occlusion across the groups. However, significantly improved blood flow in IRL-1620 compared to vehicle was observed at day 7 and the infarct size was reduced significantly in IRL-1620 treated rats compared to vehicle at 24 h and day 7, which suggested improved neuroprotection in IRL-1620 treated animals. In line with these results, we also observed improvements in neurological and motor function in IRL-1620 treated animals compared to vehicle. These results revalidated our previous findings related to the effects of IRL-1620 in the MCAO rat model and also warranted further studies to investigate the anti-apoptotic mechanism of action of IRL-1620 in the infarcted rat brains.

It has been observed that ET-1 binding to endothelial cell ET_B_ receptors *in vitro* leads to increased Akt phosphorylation at Ser473, endothelial nitric oxide synthase (eNOS) phosphorylation at Ser1179, and NO synthesis^38^. Also, overexpression of a dominant negative G-protein-coupled receptor kinase construct that sequesters βγ subunits has been shown to inhibit Akt phosphorylation and NO synthesis. While Akt phosphorylation is generally believed to occur through tyrosine kinases, these data demonstrate that the G-protein-coupled ET_B_ receptors can signal the PI3K/Akt pathway^39^. Activation of the PI3K/Akt pathway has been widely reported to participate in the protection against cerebral ischemia^40,41^ and lower levels of Ser473 phosphorylation were correlated with worsening damage associated with ischemia^42–45^. In this study, we observed transiently elevated pAkt (Ser473) levels in IRL-1620-treated MCAO animals at the early stage of ischemia, suggesting its role in mediating the neuroprotective effect of ET_B_ receptor stimulation. It has been reported that phosphorylation of Akt at Ser473 and Thr308 leads to subsequent phosphorylation of Bad and affects apoptosis^40,46^. Therefore, we assessed the expression of Bad and its phosphorylation after IRL-1620 treatment following ischemia. The total Bad expression level was significantly decreased in IRL-1620-treated animals in the occluded hemisphere compared to sham and vehicle-treated groups 7 h post MCAO, while we observed similar expression of Bad among all the groups at 24 h. We analyzed phospho-Bad in these groups and found no change at 7 h post MCAO among the groups; however, decreased phosphorylation of Bad in vehicle treated animals at 24 h post MCAO was observed. Furthermore, treatment with IRL-1620 increased the phosphorylation of Bad compared to vehicle-treated animals and reached similar levels to sham at 24 h post MCAO. It is possible that transiently increased pAkt levels in the IRL-1620 group will still play a role in modulating Bad activity and maintaining pBad levels similar to sham levels 24 h post MCAO.

Other major regulatory proteins involved in regulating the early stages of apoptosis include Bcl-2 and Bax, which are downstream targets of Akt^40,47^. It has been reported that the expression of Bcl-2 directly affects infarct volume in rodent model of cerebral ischemia^48,49^. Previous studies have demonstrated that various neuroprotective treatments may have the capacity to reduce the impact of stroke via increased expression levels of either Bcl-2 or Bcl-xL^50,51^. We observed decreased Bcl-2 expression and increased Bax expression in vehicle-treated MCAO animals. Interestingly, treatment with IRL-1620 upregulated Bcl-2 expression while downregulated Bax expression following MCAO. Bax is a pro-apoptotic Bcl-2 family protein and plays an important role in the mitochondria-mediated apoptosis pathway. It is a cytosolic protein and normally remains in the apoptotically inactive monomeric form; however, during apoptosis its conformational change causes homo-oligomerization and integration into the outer mitochondrial membrane (OMM). The insertion of Bax into the OMM leads to pore formation and release of apoptotic factors (e.g. apoptosis-inducing factor, endonuclease G, cytochrome c etc.) into the cytosol^27,52,53^. Release of these factors into cytosol may lead to caspase-independent (AIP, endonuclease G) or caspase-dependent (cytochrome C) DNA fragmentation and ultimately cell death^54,55^. Thus, integration of Bax into mitochondrial membrane is a critical step of apoptosis from where the reversal of apoptosis is highly unlikely. Therefore, detection of mitochondrial membrane bound Bax would be a better indicator of apoptosis than cytosolic Bax. We used immunofluorescence technique to detect the co-localization of Bax and mitochondria. We observed significantly higher expression of Bax in vehicle-treated animals at 24 h and day 7 post MCAO compared to sham. Treatment with IRL-1620 significantly decreased Bax expression compared to vehicle. Interestingly, when we analyzed the microscopic images for co-localization of Bax and mitochondria, we observed higher amounts of Bax co-localization with mitochondrial dye in vehicle compared to IRL-1620-treated animals. These observations indicate that IRL-1620 could modulate apoptotic signaling pathway and thus interfere with the docking of Bax to the mitochondrial membrane. Moreover, the results of the TUNEL assay showed a significant decrease in DNA end labeling (an indicator of DNA fragmentation) in IRL-1620 compared to vehicle-treated animals.

The pathophysiology of cerebral ischemia involves multiple complex pathways including vascular remodeling (angiogenesis/vasculogenesis), which plays an important role in repair and regeneration. Angiogenic substances in ischemic conditions have been shown to increase CBF as well as inhibit apoptosis^29,30^. Moreover, our previous study has demonstrated that ET_B_ receptor stimulation increases the expression of a potent angiogenic factor, VEGF, in the ischemic rat model^22^. Therefore, in the present study we have examined the blood flow at 1 h and day 7 post MCAO, using laser Doppler flow meter. A significant decrease in CBF was noted in the ipsilateral (occluded) hemisphere following MCAO as expected; however, a decrease in CBF in the contralateral hemisphere was also observed. A pressure gradient often occurs between neighboring arterial fields when there is a significant change in perfusion, particularly with the occlusion of a large vessel. This gradient subsequently results in alterations of collateral blood flow rate and direction, which may explain the observed decrease in CBF in the non-occluded hemisphere^56–58^. As we occluded the MCAO permanently, the filament remains in the artery and might cause a rapid drop in intraluminal pressure and vascular resistance, leading to a collateral circulation fail during initial hours, thereby reducing CBF in the contralateral hemisphere as well as the ipsilateral. We observed a significant increase in CBF in IRL-1620-treated animals at day 7 post MCAO. It is possible that IRL-1620 could be limiting the injury due to ischemia by improving the CBF and by inhibiting the apoptotic pathway.

**Figure 7:**
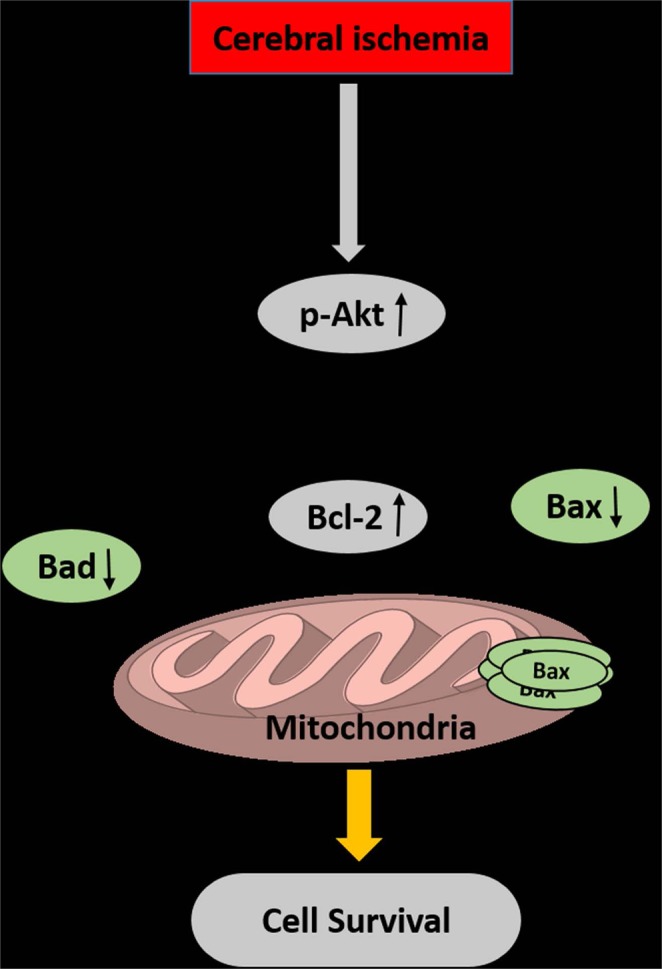
Stimulation of ET_B_ receptors by IRL-1620 can stimulate apoptotic signaling pathways which may be implicated in its neuroprotective effect.

Taken together, these results indicate that IRL-1620 improves CBF, increases the level of pAkt, which attenuates its downstream target Bad, upregulates Bcl-2 expression and interferes with Bax translocation to the OMM, thereby reducing ischemic neural cell death as observed via TUNEL assay and reducing infarct size (Fig. 7). Based on our previous as well as the present studies, we suggest that stimulation of ET_B_ receptors via IRL-1620 may serve as a multimodal therapeutic agent for the treatment of stroke, providing neuroprotection from apoptosis and oxidative stress as well as enhancing endogenous neurovascular repair mechanisms. Results of these studies support the clinical development of IRL-1620 for patients with cerebral ischemia.

## Methods

### Animals

Male Sprague-Dawley rats (Envigo, Indianapolis, IN) weighing 350–375 g were allowed to acclimate for at least 4 days before use in a room with controlled temperature (23 ± 1 °C), humidity (50 ± 10%), and light (6:00 A.M. to 6:00 P.M.). Food and water were available continuously. Animal care and use of anesthetic and surgical procedures were approved by the IACUC of Midwestern University and were performed in accordance with guidelines and regulations of the U.S. National Institutes of Health. Rats were randomly divided into three groups (Group 1: Sham, Group 2: MCAO + Vehicle, Group 3: MCAO + IRL-1620) with an n = 6/group for each assessment – infarct volume, protein estimation and TUNEL assay – at 7 h, 24 h, and day 7.

### Drugs

Ketamine (Henry Schein Animal Health, Dublin, OH, USA) was administered at a dose of 100 mg/kg, intraperitoneally (i.p.), and xylazine (Lloyd Laboratories, Shenandoah, IA, USA) was administered at a dose of 10 mg/kg, i.p. IRL-1620 [N-Succinyl-[Glu9, Ala11,15] endothelin 1] (Bachem Americas, Inc., Torrance, CA 90505) was dissolved in isotonic saline and administered at a dose of 5 µg/kg, intravenously (i.v.) via tail vein at 2, 4 and 6 h post MCAO. The dose of IRL-1620 was based on our previous studies^20–22^ and Phase I safety and tolerability studies^23^.

### Middle cerebral artery occlusion to induce focal cerebral ischemia

Induction of focal cerebral ischemia via MCAO was performed according to the method of Koizumi, *et al*.^59^ with modification of access route^60^. Rats were anesthetized with ketamine (100 mg/kg, i.p.) and xylazine (10 mg/kg, i.p.). A rectal core temperature of 37 ± 1 °C was maintained throughout the operation and recovery from surgery using the thermo-controlled base of the operating table, measured with a Cole Palmer Animal Monitoring Thermometer with colonic probe (Vernon Hills, IL, USA). With the animal in a secure supine position, a midline incision was made and the right common carotid artery, external carotid artery, and internal carotid artery were exposed. A 4.0 monofilament nylon thread (CP Medical, Portland, OR, USA) was advanced from the external carotid artery into the lumen of the internal carotid artery until a resistance was felt (~20 mm), indicating occlusion of the middle cerebral artery. The nylon filament was allowed to remain in place to create a permanent model of focal cerebral ischemia. The common carotid artery and external carotid artery were exposed in sham animals and the incision was sutured without touching the internal carotid artery^20,61^. Animals were monitored until fully recovered from anesthesia on the day of surgery, and then observed for well-being 2x/day until the end of the experiment. Mortality for the day 7 study included 2 animals in the vehicle-treated group, which were then removed from the study. These animals were then repeated in order to meet the number of animals required for statistical power at the day 7 endpoint. No mortality was observed in the 7 h or 24 h studies.

### Neurological evaluation

Animals were subjected to a neurological evaluation prior to occlusion, and at 7 h, 24 h, and day 7 post MCAO. A 6 point scale to assess neurological deficit was used, with 0 indicating no deficit and 5 representing death, as described in previous studies^21,61,62^.

### Motor performance tests

Four assessments were used to determine motor activity and coordination following MCAO. Animals were subject to blinded assessments prior to occlusion, and 7 h, 24 h, and day 7 post MCAO using a grip test, foot fault test, rota rod and spontaneous locomotor activity.

#### Grip test

The grip test consisted of a string 50 cm in length, pulled taut between two vertical supports and elevated 40 cm above a flat surface. The animal was placed on the string midway between the supports and evaluated according to a 6 point scale^21,61,63^.

#### Foot fault test

Animals were placed on an elevated grid floor with a mesh size of 30 mm for one minute to acclimate. They were then observed for one minute and evaluated for foot fault errors as described earlier^21,61,64^.

#### Rota rod

Prior to MCAO, animals were acclimated to the rotating spindle of the rota rod apparatus (Rota-Rod 47700, Ugo Basile, Italy) as described earlier. Animals were then placed on the rotating spindle for acceleration trial and the time at which they fell off was recorded in seconds^21,61,65^.

#### Spontaneous locomotor activity

Spontaneous locomotor activity was assessed using an animal activity meter (Opto-Varimex-4 Auto-Track System, Columbus Instruments, Columbus, OH). Each animal was observed for a period of 10 min in a square enclosed area equipped with infrared photocells along the X, Y, and Z axes to quantitatively measure spontaneous horizontal and vertical motion^66^.

### Cerebral blood flow

Cerebral blood flow (CBF) was monitored using Laser Speckle Contrast Analysis (LASCA) via the PeriCam PSI High Resolution System (Perimed, Sweden). The rats were anesthetized and a paramedian skin incision was performed. The subcutaneous tissue and cranial fascia were dissected to reach the skull bone. The rats were then positioned precisely 10 cm below the laser with a 1.1 cm^2^ field centered over the midline. CBF was monitored at a frame rate of 25 images/sec for 15 minutes before, and at 1 h and day 7 post MCAO. Baseline CBF readings taken 15 min prior to occlusion were compared with CBF readings taken at 1 h and day 7 post MCAO. Complete occlusion of the MCA was considered to have occurred when the infarcted hemisphere demonstrated a >40% decrease in CBF at 1 h post MCAO as compared to baseline.

### Assessment of cerebral infarct volume

Brains were removed to determine infarct volume at 7 h, 24 h and day 7 post MCAO. Animals were anesthetized with ketamine and xylazine and euthanized by decapitation. The brains were quickly removed and chilled in saline at 4 °C for 5 min. They were then cut into 2 mm thick coronal slices using a Brain Matrix (Harvard Apparatus, Holliston, MA). Sections were incubated in 2% 2,3,5-triphenyltetrazolium chloride (TTC, Sigma, St. Louis, MO) dissolved in saline for 15 min at 37 °C^67^. Infarct volumes were calculated by sampling each side of the coronal sections with a digital camera (Nikon, Melville, NY). The infarct area, outlined in white, was measured by image analysis software (Adobe Photoshop CS6). Infarct size is expressed as infarction volume in mm^3^ as the sum of infarct areas in each slice, corrected for edema^21,61^.

### Estimation of apoptotic markers

At 7 h, 24 h, and day 7 post MCAO, animals were euthanized by decapitation, and the brains were removed for western blot analysis. Brain tissues were washed in chilled saline and homogenized in RIPA buffer (20 mM Tris-HCl pH 7.5, 120 mM NaCl, 1.0% Triton X100, 0.1% SDS, 1% sodium deoxycholate, 10% glycerol, 1 mM EDTA and 1X protease inhibitor, Roche). Proteins were isolated in solubilized form and concentration was determined using Folin-Ciocalteu’s Reagent^68^. Solubilized protein (60 μg) was denatured in Laemmli sample buffer (Bio-Rad, Hercules, CA), resolved in 10% SDS–PAGE and transferred on nitrocellulose membrane (Sigma-Aldrich, St. Louis, MO, USA). The membrane was then blocked with superblock solution for 1 h at room temperature. The membranes were incubated overnight with rabbit polyclonal anti-Akt, pAkt, Bad, pBad, Bax and Bcl-2 antibodies (1:1000) (Cell Signaling Technology, Danvers, MA, USA) at 4 °C overnight, followed by incubation with goat anti-rabbit IgG, horseradish peroxidase-conjugated (HRP) secondary antibody (1:2000) (Santa Cruz Biotech., Santa Cruz, CA, USA) for 2 h at room temperature. β-actin (1:10,000; Sigma-Aldrich, St. Louis, MO, USA) was used as a loading control. The labeled proteins were visualized with SuperSignal WestPico Chemiluminescent Substrate (Thermo Fisher Scientific, Bartlett, IL) using the Kodak Gel Logic 1500 Imaging System (CarestreamHealth Inc., New Haven, CT). Protein expression was analyzed using ImageJ (NIH) software^61^.

### Immunofluorescence

To confirm western blot data and examine the sub-cellular localization of Bax in relation to mitochondria, we used immunofluorescence technique to detect the co-localization of Bax and mitochondria in the rat brain tissue after staining with anti-Bax and mitotracker dye (Nonyl Acridine Orange, Thermo Fisher Scientific, Waltham, MA). We selected this dye because of its property to enter into mitochondria independent of their membrane potential. Therefore, it is suitable to use in live cells as well as in fixed tissues. At 24 h and day 7 following MCAO, animals underwent transcardial perfusion to fix the brains. The brains were post-fixed in 50 ml of 4% PFA in NaPO4 buffer solution for 2 h, and then placed in 20% sucrose/4% PFA, pH 7.4, 50 ml/brain at 4 °C for 48 h. The brains were then sliced into 10 µm thick slices for immunofluorescent analyses at −20 °C using a cryostat (Microtome cryostat HM 505E; Walldorf, Germany). The brain sections were washed with 1X HBSS twice and incubated with 5 µM Mitotracker dye (prepared in 1X HBSS) for 30 minutes at room temperature. After washing three times with 1X PBS, permeabilization was performed with 1% Triton-x100 in PBS for 15 minutes at room temperature. Blocking was performed with 5% BSA in 1X PBS for 1 h at room temperature. The brain sections were incubated with anti-Bax antibody (1:200 diluted in 1X PBS) at 4 °C overnight. Sections were washed twice in 1X PBS and incubated with Alexa Fluor 555-conjugated donkey anti-rabbit secondary antibody (1:200, Abcam, Cambridge, MA) for 1 h at room temperature in the dark and mounted with prolong gold anti-fade reagent with DAPI (Cell Signaling Technology, Danvers, MA, USA). Fluorescence was detected using an inverted fluorescent microscope (Nikon Eclipse TiE, Melville, NY). All images for analysis were taken with the same exposure with a multi-channel ND acquisition using NIS Elements BR imaging software (Nikon Instruments, Inc., Melville, NY). Analyses was performed using NIS-Elements 3.01 imaging software from Nikon Instruments, Inc. (Melville, NY).

### Terminal deoxynucleotidyl transfer-mediated dUTP nick end labeling (TUNEL) assay

At 24 h and day 7 following MCAO, animals underwent transcardial perfusion to fix the brains. The brains were post-fixed in 50 ml of 4% PFA in NaPO4 buffer solution for 2 h, and then placed in 20% sucrose/4% PFA, pH 7.4, 50 ml/brain at 4 °C for 48 h. The brains were then sliced into 10 µm thick slices for immunofluorescent analyses at −20 °C using a cryostat (Microtome cryostat HM 505E; Walldorf, Germany). Coronal cryostat sections were processed according to the manufacturer’s instructions for TUNEL assay using Click-iT TUNEL Alexa Fluor Imaging Assay kit (Molecular Probes, Invitrogen, NY, U.S.A.). Cells exhibiting DNA fragmentation (TUNEL positive) were counted in the tissues using inverted fluorescent microscope (Nikon Eclipse TiE, Melville, NY) in a blinded fashion^61,69,70^.

### Statistical analysis

A Power Analysis was conducted using GraphPad Instat-3.1 with a beta of 0.8 and alpha of 0.05. The sample size in each group was N = 6 based upon expected change determined from results published in literature using similar procedures. Data are presented as mean ± S.E.M. Two-way analysis of variance (ANOVA) was used for intergroup comparison for behavioral data. One-way ANOVA followed by Bonferroni’s post hoc comparison test was used for intergroup comparison to evaluate infarct volume and apoptotic markers. A *P* value of less than 0.05 was considered to be significant. The statistical analysis was processed with GraphPad Prism 6.00 (GraphPad, San Diego, CA, USA).
