# Author Correction: Anti-apoptotic activity of ET_B_ receptor agonist, IRL-1620, protects neural cells in rats with cerebral ischemia

**DOI:** 10.1038/s41598-020-60114-2

**Published:** 2020-02-14

**Authors:** Seema Briyal, Amaresh K. Ranjan, Mary G. Hornick, Anupama K. Puppala, Thanh Luu, Anil Gulati

**Affiliations:** 1 Chicago College of Pharmacy, Midwestern University, Downers Grove, IL 60515 USA; 2 Chicago College of Osteopathic Medicine, Midwestern University, Downers Grove, IL 60515 USA; 3 Present Address: Pharmazz, Inc., Research and Development, Willowbrook, IL USA

Correction to: *Scientific Reports* 10.1038/s41598-019-46203-x, published online 18 July 2019

This Article contains errors in Figures 3, 4, 5, 6 and 7 where the white backgrounds have been erroneously changed to black. The correct Figures 3, 4, 5, 6 and 7 appear below as Figures 1, 2, 3, 4 and 5 respectively.

**Figure 1:**
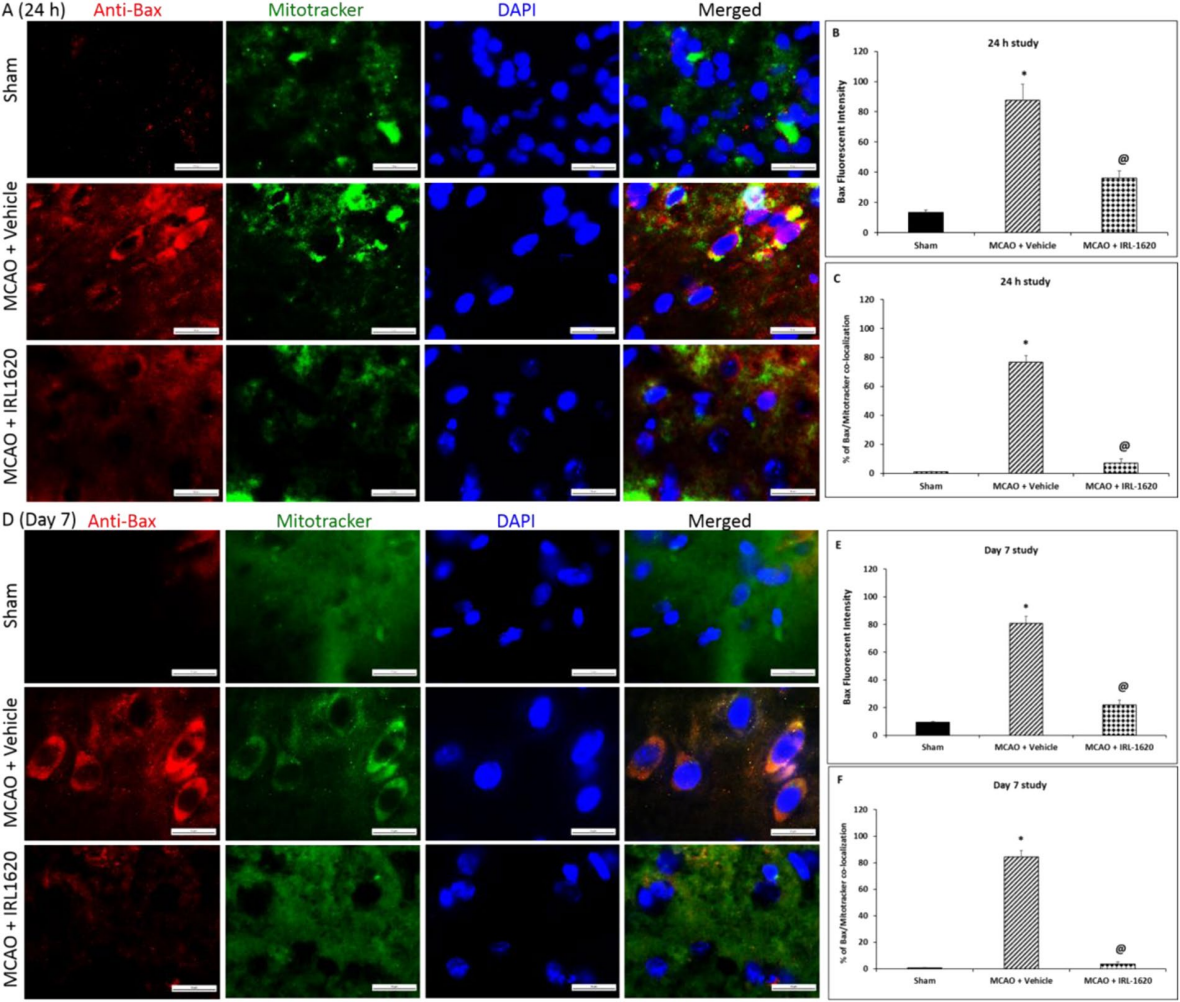
Bax translocation to mitochondria in cerebral ischemia-induced apoptosis. Bax was immuno-stained with anti-Bax^34^, and the mitochondria were stained with MitoTracker (green). The merged image indicates colocalization of Bax on mitochondria. Values are expressed as mean ± S.E.M. *P < 0.01 compared to sham, ^@^P < 0.001 compare to MCAO + vehicle.

**Figure 2:**
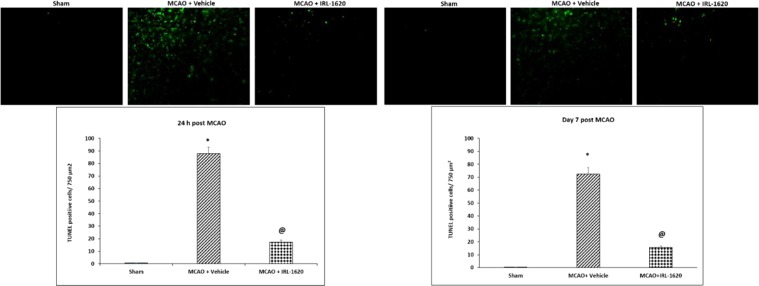
TUNEL positive cells per 750 μm^2^ in the ischemic region were detected by TUNEL staining 24 h and day 7 after MCAO. Values are expressed as mean ± S.E.M. *p < 0.0001 compared to sham; ^@^p < 0.001 compared to vehicle.

**Figure 3:**
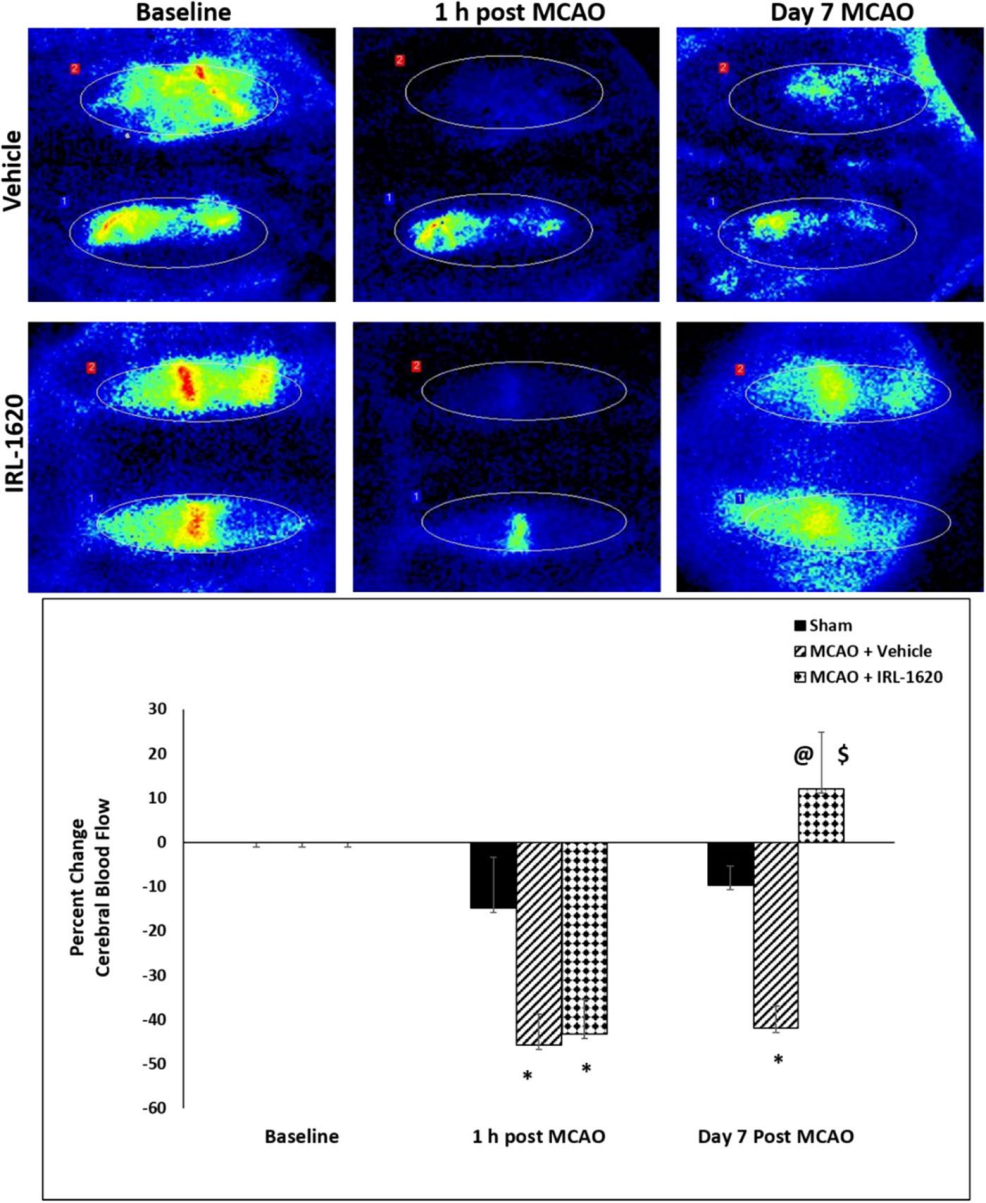
Effect of IRL-1620 on cerebral blood flow before, after and day 7 post MCAO in rat brains. Values are expressed as mean ± SEM. *P < 0.001 compared to sham; ^@^P < 0.05 compared to MCAO + vehicle; ^$^P < 0.0001 compared to IRL-1620 1 h post MCAO.

**Figure 4:**
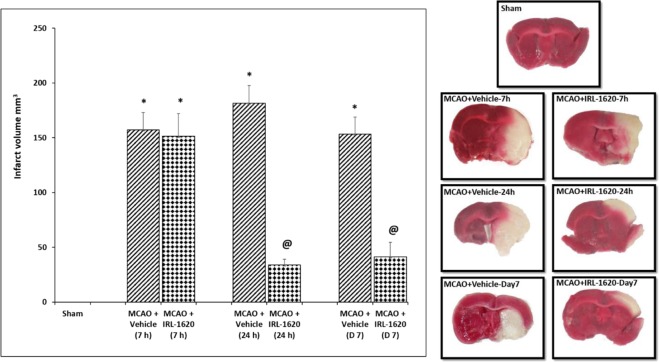
Effect of IRL-1620 on infarct volume in MCAO rats. 2 mm coronal sections of brains stained with TTC to visualize the infarct area 7 h, 24 h and day 7 post MCAO (red indicates normal tissue and white indicates infarct tissue). Values are expressed as mean ± SEM. *P < 0.001 compared to sham; ^@^P < 0.05 compared to MCAO + vehicle.

**Figure 5:**
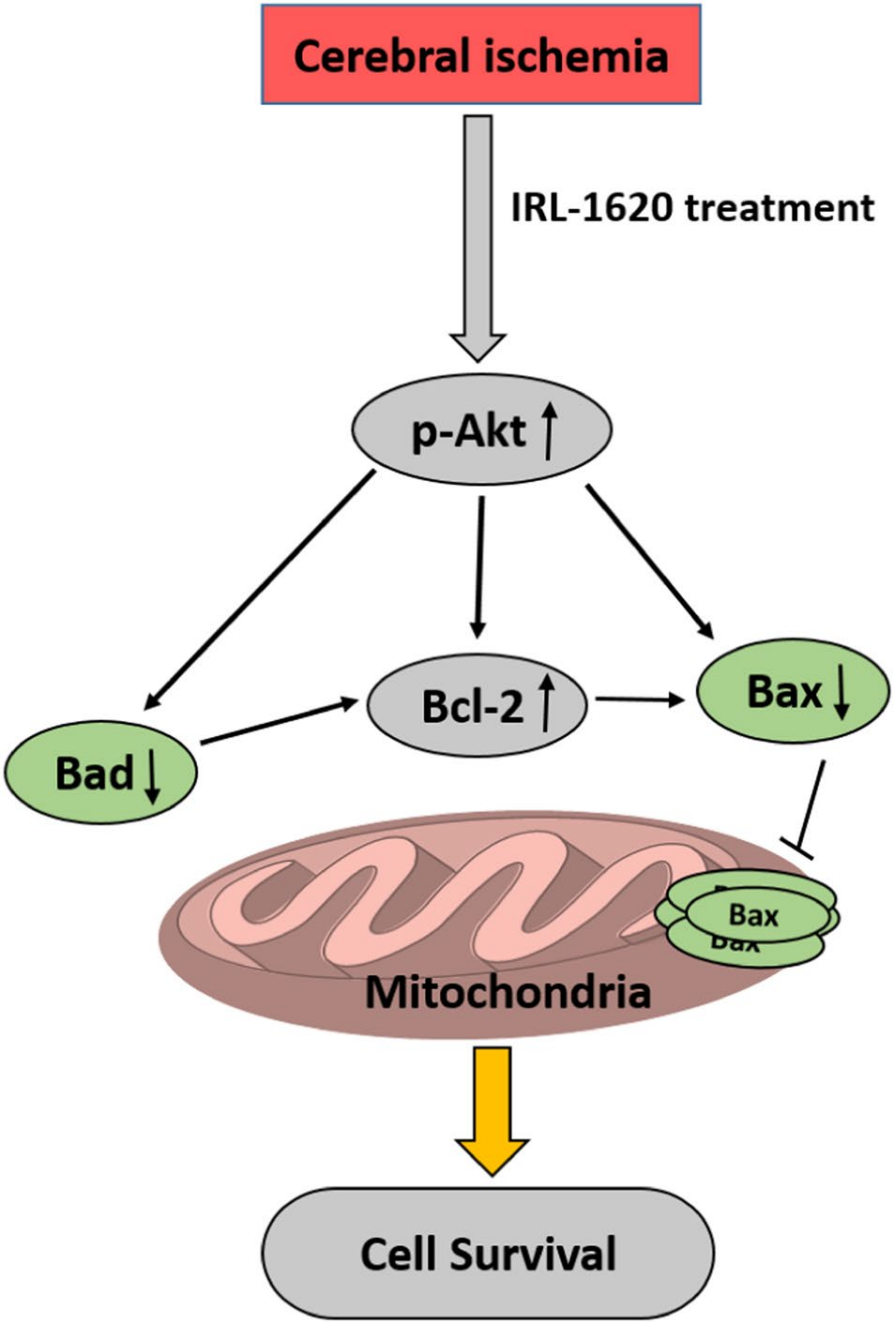
Stimulation of ET_B_ receptors by IRL-1620 can stimulate apoptotic signaling pathways which may be implicated in its neuroprotective effect.

